# Model learning to identify systemic regulators of the peripheral circadian clock

**DOI:** 10.1093/bioinformatics/btab297

**Published:** 2021-07-12

**Authors:** Julien Martinelli, Sandrine Dulong, Xiao-Mei Li, Michèle Teboul, Sylvain Soliman, Francis Lévi, François Fages, Annabelle Ballesta

**Affiliations:** INSERM UMR-S 900, Institut Curie, MINES ParisTech CBIO, PSL Research University, 92210 Saint-Cloud, France; Lifeware Group, Inria Saclay Ile-de-France, Palaiseau 91120, France; UPR “Chronotherapy, Cancers and Transplantation”, Paris-Saclay University, Faculty of Medicine Kremlin Bicêtre, Le Kremlin Bicêtre, 94270, France; UPR “Chronotherapy, Cancers and Transplantation”, Paris-Saclay University, Faculty of Medicine Kremlin Bicêtre, Le Kremlin Bicêtre, 94270, France; Côte d’Azur University, CNRS, INSERM, iBV, Nice 06000, France; Lifeware Group, Inria Saclay Ile-de-France, Palaiseau 91120, France; UPR “Chronotherapy, Cancers and Transplantation”, Paris-Saclay University, Faculty of Medicine Kremlin Bicêtre, Le Kremlin Bicêtre, 94270, France; Hepato-Biliary Center, Paul-Brousse Hospital, Assistance Publique-Hôpitaux de Paris, Villejuif 94800, France; Lifeware Group, Inria Saclay Ile-de-France, Palaiseau 91120, France; INSERM UMR-S 900, Institut Curie, MINES ParisTech CBIO, PSL Research University, 92210 Saint-Cloud, France

## Abstract

**Motivation:**

Personalized medicine aims at providing patient-tailored therapeutics based on multi-type data toward improved treatment outcomes. Chronotherapy that consists in adapting drug administration to the patient’s circadian rhythms may be improved by such approach. Recent clinical studies demonstrated large variability in patients’ circadian coordination and optimal drug timing. Consequently, new eHealth platforms allow the monitoring of circadian biomarkers in individual patients through wearable technologies (rest-activity, body temperature), blood or salivary samples (melatonin, cortisol) and daily questionnaires (food intake, symptoms). A current clinical challenge involves designing a methodology predicting from circadian biomarkers the patient peripheral circadian clocks and associated optimal drug timing. The mammalian circadian timing system being largely conserved between mouse and humans yet with phase opposition, the study was developed using available mouse datasets.

**Results:**

We investigated at the molecular scale the influence of systemic regulators (e.g. temperature, hormones) on peripheral clocks, through a model learning approach involving systems biology models based on ordinary differential equations. Using as prior knowledge our existing circadian clock model, we derived an approximation for the action of systemic regulators on the expression of three core-clock genes: *Bmal1*, *Per2* and *Rev-Erbα*. These time profiles were then fitted with a population of models, based on linear regression. Best models involved a modulation of either *Bmal1* or *Per2* transcription most likely by temperature or nutrient exposure cycles. This agreed with biological knowledge on temperature-dependent control of *Per2* transcription. The strengths of systemic regulations were found to be significantly different according to mouse sex and genetic background.

**Availability and implementation:**

https://gitlab.inria.fr/julmarti/model-learning-mb21eccb.

**Supplementary information:**

Supplementary data are available at *Bioinformatics* online.

## 1 Introduction

Clinical research communities currently advocate for more personalized and precise medicine to improve patient outcomes. To that end, innovative technologies have been designed to assess biological features in cell cultures, laboratory animals or patients. Systems medicine approaches aim to study these multi-type datasets through the design of patient digital twins (The CASyM Consortium, 2014; Wolkenhauer et al., 2014). This *in silico* version of the patient is based on mathematical models that represent the detailed physiology of key intracellular pathways driving disease evolution and treatment response. Such models are most frequently based on ordinary differential equations (ODEs). Traditionally, the structure of these models, e.g. chemical reaction networks, is inferred from an extensive review and subsequent summary of the literature by the modeler. More recently, efforts have been made to develop so-called *model learning* algorithms to assist humans in that task in order to automate model structure design. These methods have been applied for the search of gene regulatory networks or phosphoproteomic networks (Chan *et al.*, 2017; Ostrowski et al., 2016). In the context of ODE-based models, some machine learning techniques combine the use of time series data with a facilitated integration of prior knowledge (Aalto *et al.*, 2020; Huynh-Thu and Geurts, 2018). Known regulatory mechanisms or kinetic rates are directly accounted for in the equations. This simplifies the problem when dealing with large models, for which subparts are well known. This being said, none of these approaches provide quantitative insights about the inferred interactions. Indeed, the underlying kinetics between the target and the regulators are obtained in a non-mechanistic manner using e.g. boosted decision trees or gaussian processes. Consequently, dealing with datasets involving multiple related groups or individuals as can be the case in clinical trials including patients of different sex or genetic background, would not be possible. Thus, there is a need for the design of a network inference method able to handle prior knowledge in a context where quantitative patient-specific information needs to be accounted for in the inferred model.

This systems biology approach was developed in the context of circadian rhythms and chronotherapy, that consists in administering drug according to the patient’s 24 h-rhythms toward improved treatment outcomes. Diseased and healthy tissues display time-dependent variations over the 24 h span, which are called circadian rhythms (Ballesta *et al.*, 2017). The mammalian circadian timing system (CTS) is composed of a central pacemaker, the suprachiasmatic nuclei (SCN), located in the hypothalamus, which display spontaneous circadian rhythms and are themselves under the control of environmental cues such as light or socio-professional interactions, that force their period to exactly 24 h. Each cell is endowed with a molecular clock composed of approximately 15 genes, organized in regulatory feedback loops. These cellular clocks are exposed to systemic regulators aiming to synchronize cells within an organ and among the organism in order to orchestrate the body function and anticipate its needs over the day and night cycles for optimal energy management. The SCN coordinates most physiological signals toward peripheral organs which are in the form of biomechanical stresses, temperature cycles, hormonal variations (e.g. cortisol, melatonin) or nutrient exposure (Ballesta *et al.*, 2017). Rhythmic behaviors such as feeding patterns also impact the peripheral clocks in an SCN-independent fashion. However, the precise molecular interactions between clock genes and systemic regulators are not fully understood. Here we propose a model learning investigation to inform this biologically relevant issue.

Most processes of drug pharmacology display 24 h-rhythm with differences of several folds between minimum and maximum activities. Antitumor chronotherapies achieved an up-to-5-fold decrease in treatment side effects and nearly doubled antitumor efficacy compared to conventional administration of the same drug doses in cancer patients (Ballesta *et al.*, 2017). However, recent findings concluded to a large impact of patients’ sex, genetic background and lifestyle on drug optimal timing, thus highlighting the need for individualized chrono-infusion schemes to further improve treatment outcome. This need has initiated the development of eHealth platforms dedicated to the follow up of key circadian biomarkers in individuals (Kim et al., 2020). For instance, the PiCaDo platform, that integrates data from wearable sensors recording rest-activity, position and skin-surface temperature, was validated for safe home-based assessment of patient’s rhythms (Innominato et al., 2018; Komarzynski et al., 2018). Such information may be combined to measurements of key markers in blood or salivary samples, such as melatonin and cortisol, and to food diary keeping track of nutrient intake. However, there does not exist a methodology for the prediction of personalized drug timing from these patients’ circadian datasets, a challenge we aim to address.

Drug toxicities and efficacy are ultimately determined at the molecular scale by the response of gene and protein networks involved in the drug pharmacokinetics (PK) and pharmacodynamics (PD) in relevant organs (e.g. the liver for drug metabolism). Numerous of these intracellular regulatory networks are under the tight control of the cellular circadian clock (Ballesta *et al.*, 2017). Hence, the information needed to personalize chronotherapy consists in the circadian variations of proteins involved in drug PK-PD. Such detailed physiology and its temporal organization are unlikely to be completely assessed in individual patients due to the invasive nature and high frequency of the clinical measurements that would be required. As a consequence, there does not exist such clinical dataset comprising both circadian biomarkers and circadian rhythms of clock and pharmacological genes in peripheral organs in the same individuals, so that purely statistical approaches cannot be applied here. Hence, we aim to design a systems pharmacology mechanism-based approach to predict patient-specific circadian rhythms of clock genes and key pharmacological enzymes from non-invasive monitoring of circadian biomarkers.

We here rely on systems biology and systems pharmacology approaches that offer to dynamically model, through ODEs, key intracellular pathways. Model variables and parameters do have a physical meaning that is conserved across species, so that sub-model structures and parameter values can be validated in pre-clinical settings and further integrated in patient models, as in a multi-scale pipeline. Thus, we have developed our model learning approach using extensive circadian datasets available in four classes of mice (2 strains, 2 sexes) as a first step toward clinical application. After describing the available mouse datasets, we will expose our approach of model learning and then present the results obtained in terms of biological predictions.

## 2 Available data: circadian biomarkers and liver clock gene expression in four mouse classes

**Fig. 1.**
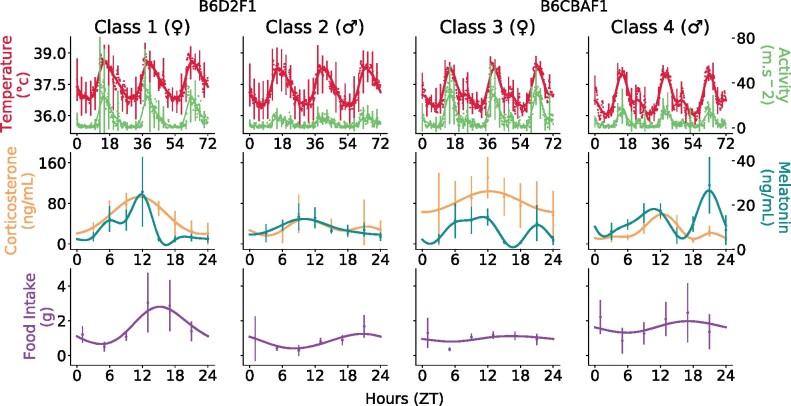
Circadian biomarkers in four mouse classes. Raw data are represented with dots (average) and error bars (standard deviations). For the sake of readability, error bars were only displayed every 2.5 h for the first line. Solid lines stand for the mean function obtained by fitting a Gaussian Process

This study aiming to identify the control of systemic regulators on the cellular circadian clock was based on extensive circadian datasets available in both male and female mice of B6D2F1 and B6CBAF1 strains (Ahowesso *et al.*, 2011; Li et al., 2013). Class 1 and 2 were defined as female and male B6D2F1 mice, Class 3 and 4 as female and male B6CBAF1 mice, respectively. For each mouse class, five systemic biomarkers were measured around the clock, which were body temperature, rest-activity, food intake, plasma corticosterone and melatonin (Fig. 1). The first two biomarkers were captured by an implanted sensor providing data every 10 min for 72 h, with up to 8 biological replicates per point (Ahowesso *et al.*, 2011). For the plasma corticosterone and melatonin, the time resolution was 3 h with 3 biological replicates (Ahowesso *et al.*, 2011; Li et al., 2000). Finally, the amount of food in a cage housing 3 mice was weighted every 4 h using a precision scale. The value measured for food intake consists of the amount of food at time *T*_1_ minus the amount of food at the next circadian time *T*_2_ (Ali and Kravitz, 2018). 3 biological replicates were used per time point. Circadian rhythms were validated using Cosinor for all classes for temperature, rest-activity and melatonin (*P *<* *0.05). Concerning corticosterone, all classes but class 2 displayed circadian rhythms (*P *=* *0.08). Food intake was predicted to display circadian variations for Class 1 and 2, only (*P *=* *0.16 and *P *=* *0.31 for class 3 and 4, respectively). Significant sex differences could be observed for instance in rest-activity profiles in terms of mesor as well as relative circadian amplitudes, although the phases were similar. Conversely, temperature profiles were virtually identical across classes. Overall, phases are well-preserved from one class to another for all biomarkers. Furthermore, mRNA circadian concentrations of the core-clock genes *Bmal1*, *Per2* and *Rev-Erbα* were measured in the mouse liver for the four classes ((Li et al., 2013) . Circadian rhythms were validated for all genes and classes (Cosinor *P *<* *0.05). Gene expressions were quite alike classwise in terms of phases. All datasets were preprocessed using Gaussian processes with a 24 h-periodic kernel (Fig. 1, (Rasmussen and Williams, 2006)).

## 3 Model learning approach

### 3.1 Accounting for direct and indirect action of systemic regulators on the clock

The five measured circadian biomarkers (rest-activity, temperature, food intake, corticosterone and melatonin) are considered as possible systemic regulators of the clock. We here focus on liver cells which do not express receptors to melatonin so that we do not anticipate any direct control of this feature on the clock. It is thus integrated in the study as a negative control. Regulators may have either immediate or time-shifted interactions with clock genes. Indeed, intermediate species are likely to be involved in the influence of these regulators on the clock. This would induce time delays as compared to the biomarkers data. For instance, temperature increase may lead to an enhanced expression of Heat-shock proteins (HSP) which then interact with clock genes (Kornmann et al., 2007). Such cascade of events would induce a phase shift between the action of the direct regulator (e.g. HSP) and the data of the corresponding biomarker (e.g. temperature). Let us assume that the regulator *z*_1_ produces the species *Z*_1_ through a linear kinetics with rate constant *k*_1_, an explicit formula is obtained for *Z*_1_ as:

(1) $$\dot{Z_{1}}(t)=k_{1}z_{1}(t)\Rightarrow Z_{1}(t)=k_{1}\int_{0}^{t}z_{1}(s)ds.$$

Hence, direct action of the five regulators are represented by the corresponding circadian biomarker data $\bar{z_{j}}$ and indirect actions are included through *integral regulators* $\bar{Z_{j}}$:

(2) $$\bar{z}={\left(\bar{z_{j}},\bar{Z_{j}}\right)}_{1\;⩽\;j\;⩽\;5}\;\text{where}\;Z_{j}(t)=\int_{0}^{t}z_{j}(s)ds.$$

*k*_1_ being incorporated into parameters of the statistical models, see below.

### 3.2 Setting a regression problem, using an ODE-based model of the liver circadian clock

In order to identify the action of systemic regulators on the cellular circadian clock, we settled for a model-based approach, which enables us to derive a mathematical expression for the approximation of this action. This approximation relies on several hypothesis described in this section. We use an ODE-based model of the mouse liver circadian clock which recapitulates the molecular interactions between clock genes and their transcription, nuclear transport and degradation [Supplementary Fig. S1-1 (Hesse *et al.*, 2021)]. Briefly, CLOCK/BMAL dimer is assumed to enhance the transcription of clock genes *Rev-Erbα*, *Rorγ*, *Per2* and *Cry1* and PER/CRY complex to inhibit this transcriptional activation. The model includes two main negative feedback loops. The first one involves the self-inhibition of *Bmal1* through the activation of its repressor REV-ERB by the dimmer CLOCK/BMAL. On the opposite, ROR whose expression is also increased by CLOCK/BMAL presence, acts positively on *Bmal1* modulation. The second feedback loop is induced by the self-repression of *Per2* and *Cry1* gene expression through the inhibition of CLOCK/BMAL transcriptional activity by the PER/CRY protein complex. In addition, REV-ERB inhibits *Cry1* gene expression, thus inhibiting its own inhibition through the modulation of PER/CRY level. In this mathematical model, the expression of gene *x* is typically described by the following differential equation:

(3) $$\frac{dx}{dt}=V_{\max}\text{Transc}(M,\gamma )-\alpha x.$$

The right term of Equation 3 accounts for gene mRNA degradation occurring at constant rate *α*. $V_{\max}$ stands for the gene transcription level in the absence of modulators. The function Transc embodies the action of modulatory species M on *x* transcription through Hill-like kinetics terms parametrized by γ. For instance, the positive action of the ROR protein on *Bmal1* transcription and the counter inhibitory part from REV-ERB action (Guillaumond *et al.*, 2005) are modeled as:

(4) $${\text{Transc}}_{\mathrm{Bmal}1}=\frac{1+\gamma_{1}{\left(\frac{\text{ROR}}{\gamma_{2}}\right)}^{\gamma_{3}}}{1+{\left(\frac{\text{REV}-\text{ERB}}{\gamma_{4}}\right)}^{\gamma_{5}}+{\left(\frac{\text{ROR}}{\gamma_{2}}\right)}^{\gamma_{3}}},$$

where *γ*_1_ is a fold transcription ratio parameter, $\gamma_{2},\gamma_{4}$ are modulation ratio parameters and $\gamma_{3},\gamma_{5}$ are Hill coefficients.

The available model represents the liver circadian organization as a dynamic purely driven by intracellular feedback loops and does not explicitly include the influence of systemic cues such as temperature or hormonal exposure which yet contribute to the liver circadian clock robustness (Ballesta *et al.*, 2017). A key question lays in the molecular links between the cellular clock and those systemic circadian regulators. Hence, they will be included in a new form of the mouse liver clock model as follows. We consider that the action of systemic regulators z on the circadian cellular clock is done by a forcing function *f*, and any feedback from the clock to the systemic regulators is neglected. Two regulations are considered as multiplicative action of the regulators on either gene transcription or gene mRNA degradation so that the dynamics of a gene *x* in an *in vivo* scenario can be written as one of the following equations:

**Hypothesis H1:**

(5) $$\begin{array}{c} \frac{dx^{\mathrm{vivo}}}{dt}=f(z)V_{\max}\text{Transc}(M,\gamma )-\alpha x^{\mathrm{vivo}}\\ \Leftrightarrow f(z)=\frac{\frac{dx^{\mathrm{vivo}}}{dt}+\alpha x^{\mathrm{vivo}}}{\text{Transc}(M,\gamma )} \end{array}$$

**Hypothesis H2:**

(6) $$\begin{array}{c} \frac{dx^{\mathrm{vivo}}}{dt}=V_{\max}\text{Transc}(M,\gamma )-f(z)\alpha x^{\mathrm{vivo}}\\ \Leftrightarrow f(z)=\frac{V_{\max}\text{Transc}(M,\gamma )-\frac{dx^{\mathrm{vivo}}}{dt}}{x^{\mathrm{vivo}}} \end{array}$$

where $f(.)\leftarrow V_{\max}f(.)$ for Equation 5 and f(.)←αf(.) for Equation 6. Incorporating $V_{\max}$ and *α* into the residual trajectories bypasses the need for any assumption on their values as they will be merged with parameters of the considered statistical models, see below.

For *Bmal1*, *Per2* and *Rev-Erbα*, *x^vivo^* can be estimated from the gene expression data ${\bar{x}}^{\mathrm{vivo}}$ available in the four mouse classes. Similarly, the five potential systemic regulators z which are rest-activity, temperature, food intake, corticosterone and melatonin can be set equal to their measurements in the mouse classes $\bar{z}$. Upon discretization over the time grid ${\left\{t_{i}\right\}}_{1\;⩽\;i\;⩽\;N}$, at which the Gaussian processes used for data preprocessing are evaluated, Equations (5) and (6) can be transformed as:

(7) $$f(\bar{z}(t_{i}))\approx \frac{\frac{\Delta {\bar{x}}^{\mathrm{vivo}}(t_{i})}{\Delta t_{i}}+\alpha {\bar{x}}^{\mathrm{vivo}}(t_{i})}{\text{Transc}(M,\gamma )}:=y(t_{i})\;\;H1$$

 

(8) $$f(\bar{z}(t_{i}))\approx \frac{V_{\max}\text{Transc}(M,\gamma )-\frac{\Delta {\bar{x}}^{\mathrm{vivo}}(t_{i})}{\Delta t_{i}}}{{\bar{x}}^{\mathrm{vivo}}(t_{i})}:=y(t_{i})\;\;H2$$

Each function *y* is called a **residual trajectory**. The goal of the study is to identify all possible functions *f* that would properly fit all residual trajectories *y*, given the systemic biomarkers measurements in the four mouse classes.

We now define a model learning problem. For the sake of simplicity, we will study the case where systemic regulators only act on either the transcription or the degradation of a single gene. This gene is either *Bmal1*, *Per2* or *Rev-Erbα* for which we have mRNA level data. For each of the six scenarios (3 genes, action on transcription or degradation), let us consider the following learning samples, for a given class of mice

$$\left\{\left(\bar{z}(t_{i}),y(t_{i})\right),\;i\in [[1,N-1]]\right\}.$$

The problem of finding the optimal functions *f* can be addressed in a regression setting, by solving

(9) $$\underset{\hat{f}\in F}{\text{argmin}}\;\frac{1}{N-1}\sum_{i=1}^{N-1}{\left(y(t_{i})-\hat{f}(\bar{z}(t_{i}))\right)}^{2}$$

for a given family of estimator functions F, such as linear functions or tree-based functions.

$\bar{z}(t_{i})$ are given by the datasets on the circadian rhythms of the five regulators in the four mouse classes so that the principal issue is now to compute the residual trajectories *y*. They are computed by Equations (7) and (8) which includes: (i) ${\bar{x}}^{\mathrm{vivo}}$ gene expression which are set equal to mouse liver mRNA levels of either *Bmal1*, *Per2* and *Rev-Erbα*; (ii) parameters *α*, $V_{\max}$ and γ which are unknown at this stage, (iii) time-resolved concentrations of the modulatory species **M** for which no data is available. To estimate the needed parameters and circadian profiles of modulators, we will investigate the circadian clock of liver cells cultured *in vitro*, that is under constant influence or complete absence of the five whole-body regulators.

### 3.3 A model of the *in vitro* liver cellular circadian clock

**Fig. 2.**
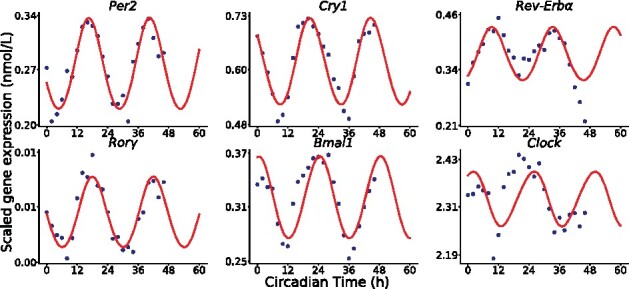
Best-fit of the *in vitro* cellular clock model (red curves) to mRNA levels of six clock genes measured in MMH-D3 cell culture (blue dots)

We leveraged time-resolved mRNA expression of six clock genes measured in immortalized MMH-D3 mouse hepatocytes using microarray technology (Atwood *et al.*, 2011). This cell line is often used as a surrogate for healthy hepatocytes. Cells were cultured in standard conditions in which they are exposed to constant temperature and access to nutrients, in the absence of mechanical stress, melatonin or corticosterone addition. Under these *in vitro* conditions, the influence of all regulators are constant over time so that gene expression can be expressed by Equation 3. The existing model of the *in vivo* mouse liver clock can thus be used to represent the *in vitro* circadian clock, yet after parameter adaptation based on cell culture data in which the clock is not under rhythmic controls. The MMH-D3 gene expression datasets were used to adjust the parameters of the *in vitro* clock model starting from estimates of the in vivo model (Hesse *et al.*, 2021). Parameter estimation is described in Supplementary File S2. RT-qPCR data from primary mouse hepatocytes culture were used to scale microarray intensities to obtain absolute values of mRNA intracellular concentrations, as required for modeling purpose (Feillet *et al.*, 2016). The fitted *in vitro* model succeeded in capturing the oscillatory behavior of the six clock genes (Fig. 2). A total of 10 optimal parameter sets were obtained from different runs of the optimization algorithm, both leading to the same reasonable fit of the data. The result can then be thought of as the cellular clock contribution isolated from the rhythmic influence of the systemic regulators. It will be used to identify specific regulators of the cellular clock in the *in vivo* setting.

### 3.4 Computing residual trajectories for the *in vivo* scenario using the *in vitro* clock model

In Section 3.2, we derived an expression for the approximation of the action of systemic regulators on the cellular circadian clock under **H1** or **H2**. Equation 9 formulates the problem in a regression setting, which requires the computation of the residuals trajectories *y*. The latter necessitates parameter values for $\alpha ,V_{\max}$ and γ, as well as the concentrations of the modulatory species M (Equations 7 and 8): REV-ERB and ROR for *Bmal1*, CLOCK/BMAL and PER/CRY for both *Per2* and *Rev-Erbα*.

While the adjustment of our circadian clock model to *in vitro* data provided estimates for these quantities, one can question their reliability in the *in vivo* setting. Given that, we have decided to identify leading systemic regulators based on the prediction of **multiple** residual trajectories obtained by varying parameter values of the *in vitro* model. This reduces the dependence of future inference on these estimates, thus ensuring the robustness of the method as functions of systemic regulators *f* would have to be optimal for numerous different liver clocks. Let θ be the model parameter vector. For selected coordinates *j*, we apply an additive Gaussian noise to *in vitro* values as follows:

**Hypothesis H3:**

(10) $$\theta_{j}^{\mathrm{vivo}}=\theta_{j}^{\mathrm{vitro}}+\epsilon \;\text{where}\;\epsilon ∼N\left(0,\frac{\theta_{j}}{\sigma }\right)$$

with *σ* a scaling factor, in practice set to 10. The relevant coordinates *j* are composed of two sets. The first set corresponds to the parameters $\alpha ,V_{\max}$ and γ involved in Equations (7) and (8). These parameters are different for each gene *Per2*, *Bmal1* and *Rev-Erbα*. The second is the set of model parameters that have the greatest impact on the time-concentration profile of modulator species M. These are best suited to make the modulators deviate from their *in vitro* concentrations. They were determined for each species through global sensitivity analysis in which outputs are defined as the circadian mean, amplitude or phase of the temporal profile of M [Supplementary Fig. S2-1 (Sobol, 2001)]. For each of these characteristics *l* and each modulator *m*, we selected the parameter set $P_{m,l}$ comprised of the *p* most sensible parameters according to Sobol sensitivity indices. Then, the intersection of $P=\underset{l,m}{\cap }P_{l,m}$ was computed. *p* was chosen such that #P=5 where # is the cardinal of a set. Among these parameters were found 3 degradation parameters for *Bmal1*, *Clock* and CLOCK/BMAL_*N*_ as well as 2 cytoplasmic protein production parameters for CLOCK_*C*_ and BMAL_*C*_. All selected sensible parameters were related to the CLOCK/BMAL loop.

**Fig. 3.**
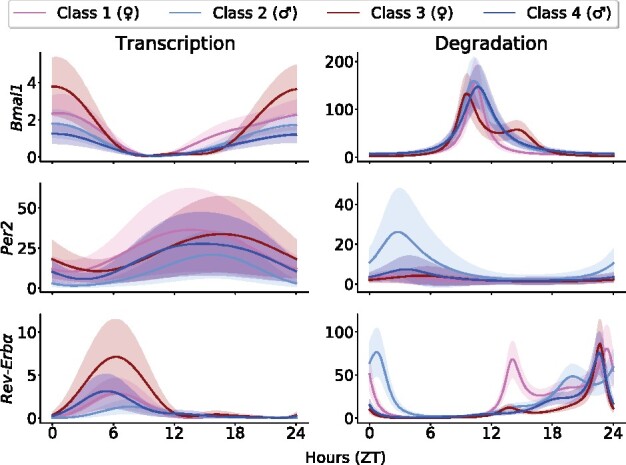
Mean and standard deviation of the selected residual trajectories obtained for each clock gene. Left (resp. right) panels under **H1**–**H3** (resp. **H2**–**H3**)

Under **H3**, additive gaussian noise is applied to each of the 10 *in vitro* parameter sets and fed to the model to compute corresponding clock variables time profiles. Considering multiple optimal parameter sets allows us to reduce the parameter uncertainty related to the lack of constraints. Selection criteria are applied in order to only select realistic clocks: (i) variable concentrations outputted should be periodic with period between 20 and 28 h, and display relative amplitude above 5%, (ii) the phase difference between the nuclear variables REV-ERB and ROR, and between PER/CRY and CLOCK/BMAL complexes should be larger than 6 h, as this two couples are made of variables that play antagonist roles (Ko and Takahashi, 2006). If all these criteria are met, the model simulation and its associated parameter set are kept and the corresponding trajectory from Equation 7 or Equation 8 is computed, using the perturbed parameter vector. This procedure is repeated until *n* trajectories are obtained, for each mouse class and gene. In practice *n* was set to 2000. Inter-class differences of circadian amplitudes and phases could be observed between the trajectories generated for each of the four mouse classes as a result of variations present in the clock gene expression data (Fig. 3).

### 3.5 Identifying action of systemic regulators as a linear regression problem

The most straightforward way to solve the problem evoked in Equation 9 is to compute an estimator of *f* thanks to linear regression. This is biologically meaningful as chemical reactions can often be written using the law of mass action that assumes linear kinetics. Besides, estimators provided in this case are easy to interpret as the contribution of a regulator *z_j_* is represented with a weight *β_j_*:

(11) $$\hat{f}(\bar{z}(t_{i}))=\sum_{j}\beta_{j}{\bar{z}}_{j}(t_{i}).$$

We assume the same model structure, i.e. active regulators for all mouse classes. Only weights β can vary across mouse strains and sexes. From a biological point of view, this is equivalent as saying that the involved regulators are the same whatever the mouse category, although the strength of their influence may vary classwise. For the sake of simplicity, any model containing both a regulator and its corresponding integral regulator is ruled out. This constraint ensures that in a model, a systemic regulator has only one way to act on the gene: either directly or indirectly. Thus, a regression model can include at most 5 terms. To select the first term, 10 choices are possible, then 8, then 6, etc since once a regulator is chosen, its associated integral regulator cannot be selected for the current model. We end up with (10×8×6×4×2)/5!=32 possible models involving exactly five regulators. The general formula below shows that there is a total of 242 models when including one to five regulators.

(12) $$\sum_{r=1}^{5}\frac{1}{r!}\prod_{j=5-r+1}^{5}2j.$$

Considering that there are *n* residual trajectories $y_{k}^{\left(c\right)}$ for each of the four classes, the learning samples become:

$$\left\{\left({\bar{z}}^{\left(c\right)}(t_{i}),y_{k}^{\left(c\right)}(t_{i})\right),\;i\in [[1,N-1]],\;c\in [[1,4]],k\in [[1,n]]\right\}.$$

For each class *c*, let us define the loss of a given model $\hat{f}$ parametrized by $\beta_{k}^{\left(c\right)}={\left(\beta_{k,j}^{\left(c\right)}\right)}_{j\in [[1,10]]}$ applied to the class regulator data ${\bar{z}}^{\left(c\right)}$ against trajectory $y_{k}^{\left(c\right)}$ as:

(13) $$\ell (y_{k}^{\left(c\right)},{\bar{z}}^{\left(c\right)},\beta_{k}^{\left(c\right)}):=\frac{1}{N-1}\sum_{i=1}^{N-1}{\left(y_{k}^{\left(c\right)}(t_{i})-\sum_{j}\beta_{k,j}^{\left(c\right)}{\bar{z}}_{j}^{\left(c\right)}(t_{i})\right)}^{2}.$$

The total error associated to this model is defined as the average of the errors of each residual trajectories across the four classes. It is computed as,

(14) $$E\left(y,\beta ,\bar{z}\right):=\frac{1}{4n}\sum_{c=1}^{4}\sum_{k=1}^{n}\;\underset{\beta_{k}^{\left(c\right)}}{\min}\;\ell \left(y_{k}^{\left(c\right)},{\bar{z}}^{\left(c\right)},\beta_{k}^{\left(c\right)}\right).$$

Finally, to allow comparison of errors and coefficients for different trajectories, both the inputs and outputs of the regression problem are standardized with zero mean and standard deviation one. Therefore the loss between a trajectory *y_k_* and an empty model is 1. One can see this value as an upper bound for the performance of a model, providing an assessment of the goodness of fit.

### 3.6 Regulator importance through Shapley values

An important question in an inference setting is to determine the precise set of relevant features in terms of prediction. Here, the fact that we deal with only ten features coupled with the low complexity cost of linear regression tolerates an exhaustive search over the whole regulators model space. Consequently, our inference considers large linear regression models as a first step and focus on smaller models thereafter. The first step of our method to identify relevant regulators for each clock gene uses Shapley values. Shortly, Shapley values stem from Game Theory and allocate to each feature *z_j_* a value $\phi_{j}$ that represents the effect of including that feature on model predictions. It is computed as the following weighted average:

(15) $$\phi_{j}=\sum_{S\subseteq F\backslash \{j\}}\frac{\#S!\left(\#F-\#S-1\right)!}{\#F!}\left({\hat{f}}_{S\cup \{j\}}({\bar{z}}_{S\cup \{j\}})-{\hat{f}}_{S}({\bar{z}}_{S})\right),$$

where *F* is the set of all feature indices, *S* a subset of *F* and **z**_*S*_ the vector of features with indices in *S*. Since the effect of *z_j_* depends on other features, the model differences are computed for all possible subsets of features (Peters, 2015). This approach was recently extended to handle any machine learning model such as tree-based models or neural networks (Lundberg and Lee, 2017). For linear models, one can derive a simpler formula: $\phi_{j}(t_{i})=\beta_{j}{\bar{z}}_{j}(t_{i})$.

We computed Shapley values for all possible regulator models involving 5 features, which is the maximum size of the model if excluding concomitant direct and indirect action of the same regulator. From Equation 12, there are 32 such admissible subsets of regulators **z**_*S*_. We call *I* the set containing all possible indice subsets *S* of cardinal 5, excluding those containing indices of direct and indirect actions of the same regulator. Pipeline 1 (Supplementary File S2) shows the procedure to compute the mean absolute Shapley values.

## 4 Results

### 4.1 Action of systemic regulators on clock gene transcription

Our first aim is to investigate possible actions of systemic regulators on the transcription of the three clock gene for which we have mRNA data: *Bmal1*, *Per2* and *Rev-Erbα*. Thus, in this section, we consider action of regulators in the form of **H1** and residual trajectories are computed under **H3** (Fig. 3, left column).

**Fig. 4.**
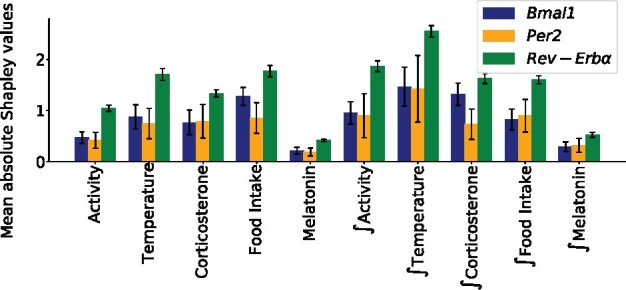
Mean absolute Shapley values for all features and each gene under **H1**–**H3**. Standard deviations computed across residual trajectories

The importance of each regulator was assessed through the computation of Shapley values for each possible linear estimator $\hat{f}$ (Fig. 4). One should notice from Pipeline 1 (Supplementary File S2) that these values are averaged across mouse classes, residual trajectories and time points. Remarkably, the lowest score is achieved by Melatonin and its indirect version ∫Melatonin, for all three clock genes. This means that according to the Shapley values metric and based on linear regression models, the melatonin is the least relevant contributor to the prediction of the trajectories *y*. This is in agreement with biological knowledge and thus provides a partial validation of the approach. Conversely, Shapley values yielded as leading regulator ∫Temperature for all three genes, advocating for a strong effect of temperature cycles on the cellular clock, yet through indirect actions involving an intermediate species.

**Fig. 5.**
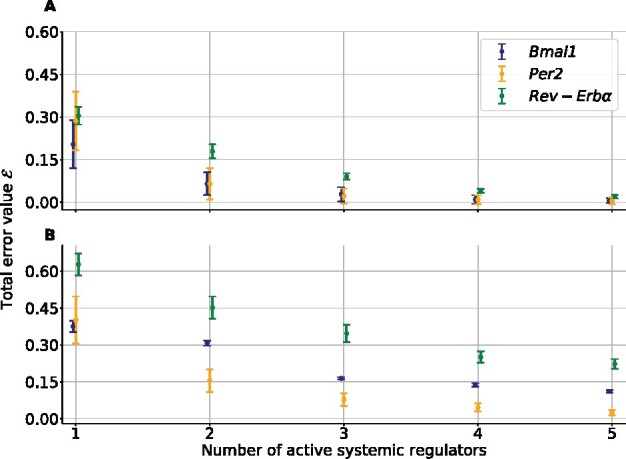
For each gene, the total error of the best-fitting model depending on its number of non-zero terms is reported for Transcription (**A**) and Degradation (**B**). Standard deviations are taken across residual trajectories

**Fig. 6.**
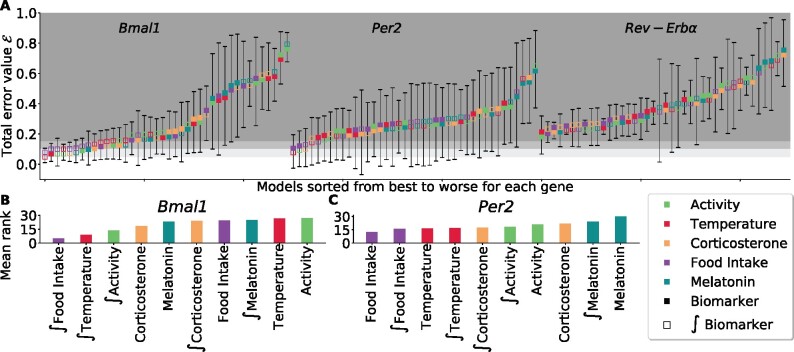
(**A**) Total error for each of the 40 2-term models representing a systemic control on either *Bmal1*, *Per2* or *Rev-erbα* transcription. Means and standard deviations were computed across residual trajectories under **H1**–**H3**. Colors indicate regulators involved in each model, with the top square referring to the dominant regulator. Areas defined by different shades of gray refer to thresholds of total error equal to 0.05, 0.1 and 0.15. (**B–C**) Mean rank of regulators among the 40 2-term models, from lowest to highest, for models impacting *Bmal1* or *Per2* transcription

While Shapley values give a coarse-grained ranking of the regulators, another level of granularity can be achieved. As mentioned earlier, the small dimension of the problem allows for an exhaustive search of all possible linear models. Under the constraint that no regulator is found twice in the same model with both a direct and indirect action, there are 242 models (Equation 12). For each gene, Figure 5 displays the total error E of the best model across residual trajectories, involving from 1 to 5 regulators. Model overfitting was investigated as follows. For each residual trajectory, time points were shuffled and divided in 4 folds on which cross validation was performed. A close agreement between training and testing total errors was found indicating that overfitting was not an issue for any considered number of systemic regulators (Supplementary Fig. S2-2). For each gene, the total error of the best-fitting model depending on its number of non-zero terms is reported under (H1–H3). Timepoints were shuffled and divided in 4-folds on which 4-fold cross validation was performed (Fig. 2). As expected, the total error decreased as terms were added to the models. The slope was found to be the steepest when moving from 1-term models to 2-term models for all genes, demonstrating the superiority of the latter in terms of balance between degrees of freedom and goodness of fit. Furthermore, for *Bmal1*, best 1-term, 2-term and 3-term models were nested, thus Fisher test could be applied. These models were ∫Temperature, ∫Temperature + ∫Food Intake and finally ∫Temperature + ∫Food Intake + ∫Activity. The 2-term model, was found to be significantly better than the 1-term and the 3-term model (*P *<* *0.05). Hence, we now focus on 2-term models which were all fitted to residual trajectories (Fig. 6). For each gene, there exists exactly $\frac{10\times 8}{2!}=40$ such models. For each model, the dominant term is defined as the regulator with indice *j* maximizing the following quantity: $\frac{1}{4n}\sum_{k=1}^{n}\sum_{c=1}^{4}|\beta_{k,j}^{\left(c\right)}|$.

The best model including a control of *Rev-Erbα* transcription achieved a poor fit to data with a total error of 0.2 which led us to discard all models for *Rev-Erbα* (Fig. 6, Supplementary Fig. S2-3). For both *Bmal1* and *Per2*, one can observe a large preponderance of the regulators food intake and temperature among the top ranked models (Fig. 6). These regulators end up with the lowest mean ranks across all systemic regulators, though only through an indirect action for *Bmal1*. For *Per2*, temperature ends up being the most present systemic regulator, involved, through direct or indirect action, in 6 out of the 10 best-performing models. This is consistent with ∫Temperature having the highest Shapley value for this gene (Fig. 4). Moreover, this finding is in agreement with the observation of an effect of the temperature on *Per2* transcription through Heat Shock Proteins reported in (Kornmann *et al.*, 2007) and provides a form of validation of our approach.

Overall 2-term models fitted for each gene, melatonin first rankings as a leading biomarker were found to be quite high: 28th, 22th and 20th for *Bmal1*, *Per2* and *Rev-Erbα*, respectively. This comes as further validation of this approach as melatonin is included here as a negative control since liver cells do not express its receptors.

### 4.2 Action of systemic regulators on clock gene mRNA degradation

In this section, we search for possible actions of the systemic regulators on clock gene mRNA degradation, assuming **H2**–**H3** hold. Figure 3 (right column) shows the residual trajectories computed with the method described in Section 3.4. These time profiles appear strongly non-linear, with sharp peaks for *Bmal1* and *Rev-Erbα* as a result of the division by the clock gene concentrations which are close to zero for certain circadian time window. Such shapes suggest that a systemic regulation of clock gene mRNA degradation would lead to an unstable control that would explode during some interval of the 24 h span. This type of behavior is unlikely to derive from the realization of natural biological processes which mostly produce robust patterns over time. Consequently, the same analysis as in the transcription case yielded to the exclusion of all models. First, for *Bmal1*, a minimum of three to four terms is necessary to achieve a proper fit of the residual trajectories, with respective total errors of 0.16 and 0.14 (Fig. 5B). In the case of *Rev-Erbα*, even 5-term models are far from producing reasonable fits, with a total error of 0.22 for the best 5-term model. For *Per2*, as in the transcription case, the slope of the total error was found to be the steepest when moving from 1-term models to 2-term model, so that all models with number of terms greater than 2 are rejected. However, with a total error of 0.31, 0.19 and 0.46 for *Bmal1*, *Per2* and *Rev-Erbα* respectively, there is no 2-term model providing a good fit of the trajectories. Total errors of all 2-term models are presented in Supplementary Figure S2-4. Overall, we conclude under **H2**–**H3**, that there is no admissible models involving a linear action of the regulators on clock gene mRNA degradation.

### 4.3 Mouse class differences

**Fig. 7.**
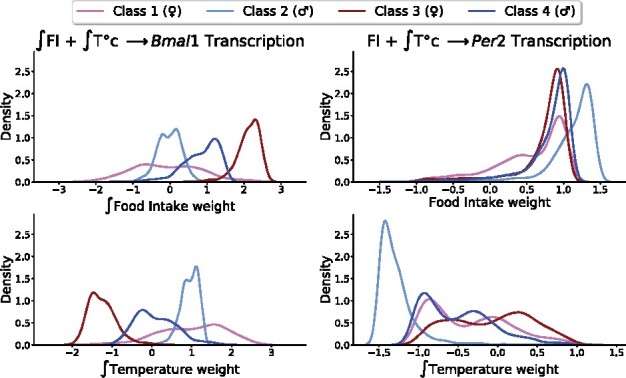
Density plot of the coefficients of the best 2-term model for *Bmal1* and *Per2*, computed across residual trajectories for each mouse class. FI: Food Intake, T°C: Temperature

As data for four mouse classes (2 strains, 2 sexes) are available, we can investigate the effect of sex and genetic background on the regulators action. Indeed, one perk of linear models is their simplicity when it comes to providing explanations: the impact of a feature on the prediction is determined by the weight associated to this feature. This enables the study of mouse class differences in terms of regulator weights. Weight distributions were estimated for each mouse class from best-fit parameters obtained across all trajectories through kernel density estimation (Fig. 7). Using all 2-term models for both *Bmal1* and *Per2* under **H1**–**H3**, we performed two-way ANOVA, asking whether or not genetic background or sex is statistically significantly impacting regulator weights. In that event, the values of regulator weights in a given model, obtained by fitting each residual trajectory, are considered as realizations of a random variable. For *Bmal1* (resp. *Per2*), 38 (resp. 37) out of 40 models agreed on the statistically significant influence of sex and genetic background on the extent of regulators influence (*P *<* *0.05). Interactions between both factors were also found to account for differences in regulators weights in 38 (resp. 37) models for *Bmal1* (resp. *Per2*). Models failing to uncover statistically significant differences were all associated with a total error above 0.2.

This finding matches the fact that circadian rhythms display sex differences in mice and in humans (Ballesta *et al.*, 2017). Moreover, previous findings demonstrated different optimal timing of the anticancer drug irinotecan in these four mouse classes (Li *et al.*, 2013).

A closer look to the weight distributions is given for *Bmal1* and *Per2*’s best fitting model (Fig. 7). Interestingly, large inter-class differences can be found for the regulator weights. The best 2-term model integrating a control of *Bmal1* transcription, involves the joint action of Food intake and Temperature, probably through intermediate species. Food intake appears to act mostly negatively on Bmal1 transcription in female and male B6D2F1 mice (Classes 1 and 2) and positively in B6CBAF1 mice (Classes 3 and 4). For Temperature, the exact reverse situation is observed. For each mouse strain, sex-specific differences are also present in the distribution modes and standards deviations, Class 1 displaying the largest variability across trajectory best-fit parameters. Next, the best model targeting *Per2* expression involves a direct positive regulation of the gene mRNA transcription by Food Intake and an indirect mostly negative influence of temperature for all mouse classes. The distributions of Food Intake weight present different shapes for Class 1 and 2, with a higher mode for Class 2, while being analogous for Class 3 and 4. Regarding the indirect action of Temperature, the distribution for Class 2 is almost set apart from the others with a lower mode, hence a stronger negative impact. The fact that parameter distributions can have classwise opposite modes raises a few questions as this would imply that systemic regulators could act either positively or negatively on gene transcription across mouse classes. As a start, model identifiability was assessed by means of profile likelihood, a method determining practical and structural identifiability (Raue et al., 2009). Supplementary Figures S2-5 and S2-6 show that the parameters of the best 2-term models for *Bmal1* and *Per2* are indeed identifiable for each class. Subsequently, similar systemic regulator weight signs across classes was enforced and models which did not initially meet this constraint, i.e. 23 for *Bmal1* and 32 for *Per2*, were re-optimized. A 1.5-fold average increase in total errors from unconstrained to constrained optimization was found (Supplementary Fig. S2-7). Altogether, these findings demonstrate that models with opposite signs were reasonable and better fit data than constrained models.

## 5 Discussion

We have presented a model learning methodology to identify systemic regulators of the peripheral circadian clocks. The theoretical approach comprises two key steps. The first step relied on the integration of extensive prior knowledge on the mammalian circadian timing system into an ODE-based circadian clock model. The comparison of this calibrated model with available circadian datasets allowed the derivation of an approximation for the action of the regulators on the clock in the form of residual trajectories. In a second step, using a linear regression framework, the task of inferring systemic regulators of the clock was interpreted as a model selection problem. The latter involving a small number of features, an exhaustive exploration of the regulator model space could be performed. Thus, we used Shapley values to draw inference on the importance of each regulator from large regression models and acquired a more fine-grained understanding with smaller models afterwards.

Our approach produces explainable linear models that mechanistically represent the action of the measured regulators on clock genes in two mouse strains. The focus was given to five regulators for which measurements were accessible: biomechanical stresses (derived from rest-activity), body temperature, nutrient exposure (derived from food intake), plasma melatonin and corticosterone. Given the available mRNA data, we were able to investigate systemic regulation of *Bmal1*, *Per2* and *Rev-Erbα* mRNA transcription or degradation. Models involving a modulation of mRNA degradation were all rejected, as well as those impacting *Rev-Erbα* transcription. Hence, all admissible models involved a regulation of either *Bmal1* or *Per2* transcription. Temperature was found to affect *Per2* transcription in an indirect manner, which was in line with temperature dependency of the expression of HSPs that interact with clock genes (Kornmann *et al.*, 2007). Similarly, melatonin which was included as a negative control was not involved in the best models. Lastly, the large predominance of food intake in the best fitting models agreed with recent experimental findings (Greenwell *et al.*, 2019). Indeed, modulating meal timing and composition impacts liver clock genes time profiles as, for instance, dampened oscillations were shown in mice subjected to high-fat-diet, whereas time restricted high-fat-diet restored regular circadian rhythms (Hatori *et al.*, 2012; Li et al., 2010). Arrhythmic feeding does not cause liver clock genes to lose oscillations in mice, a behavior which is well reproduced by our models (Greenwell *et al.*, 2019). A subsequent step would be to study the precise molecular mechanisms linking energy metabolism and the clock which requires the design of dedicated systems biology frameworks (Woller et al., 2016). Next, our approach assumes independence of the systemic regulators while this may not be the case for all of them. However, independence of temperature and food intake seems to have been validated in experiments, where different feeding patterns led to similar temperature profiles in mice (Greenwell *et al.*, 2019). Lastly, classwise opposite action of the systemic regulators was found to be necessary to ensure reasonable fit of the trajectories. Biologically speaking, differences in influences of regulators are plausible. It may imply that a systemic regulator activates different regulatory pathways for each mouse class as a consequence of different gene expression levels. For instance, it was recently found that ubiquitin associated pathways regulated the cellular clock only in female and not in male mice (Mekbib et al., 2020).

The theoretical approach developed here could be extended to handle more complex model learning scenario. We have focused on the identification of systemic regulators on gene mRNA degradation and transcription, the latter also being the starting point of gene regulatory network learning algorithm such as Dyngenie3 (Huynh-Thu and Geurts, 2018). In our case, conditionally to the availability of additional data on other species present in the clock model (proteins and protein complexes), this method could be applied to search for systemic regulations on any process included in the ODEs (e.g. nuclear translocation or protein production). For larger problems, exhaustive model search could be replaced by machine learning methods like sparse multi task regression, to leverage class information while aiming at finding a parsimonious set of optimal predictors (Lozano and Świrszcz, 2012). Finally, non-linear models can be searched for with sparse regression tools (Brunton *et al.*, 2016).

As a perspective, the best models inferred from this study will be integrated back in our ODE-based clock model and parameters will updated based on available data. The validated models will then be tested in dedicated preclinical experiments. Such an approach has been successfully employed using a small number of ODE-based models and allowed discovering new molecular interactions between clock genes and the protein p53 (Gotoh *et al.*, 2016). The next step will be the scaling of the model for humans in order to predict molecular clocks from the measurements of circadian biomarkers using wearable technologies. This will shortly be possible thanks to the availability of clinical datasets including both clock gene expression in the oral mucosa and longitudinal measurements of circadian biomarkers in the same individuals. Such human model of the circadian timing system could then be connected to drug chronoPK-PD models to derive patient-specific optimal timing (Ballesta *et al.*, 2017).

## Supplementary Material

btab297_Supplementary_Data
