# Supplementary material for: Model learning to identify systemic regulators of the peripheral circadian clock
Source: Bioinformatics. 2021 Jul 12;37(Suppl 1):i401–9. doi: 10.1093/bioinformatics/btab297 (PMC8557835; doi:10.1093/bioinformatics/btab297)
Supplement: btab297_Supplementary_Data [file btab297_supplementary_data.zip › MARTINELLI.116.sup.1.pdf]

Supplementary File S1: Mammalian cellular circadian clock model

Julien Martinelli, Sandrine Dulong, Xiao-Mei Li, Michèle Teboul, Sylvain Soliman, Francis Lévi, François Fages, Annabelle Ballesta

Contents

1 Mathematical Description of the Quantitative Core-Clock Model 2
1.1 Variable Names . . . 2
1.2 Model equations . . . 2
1.3 Model parameters . . . 5

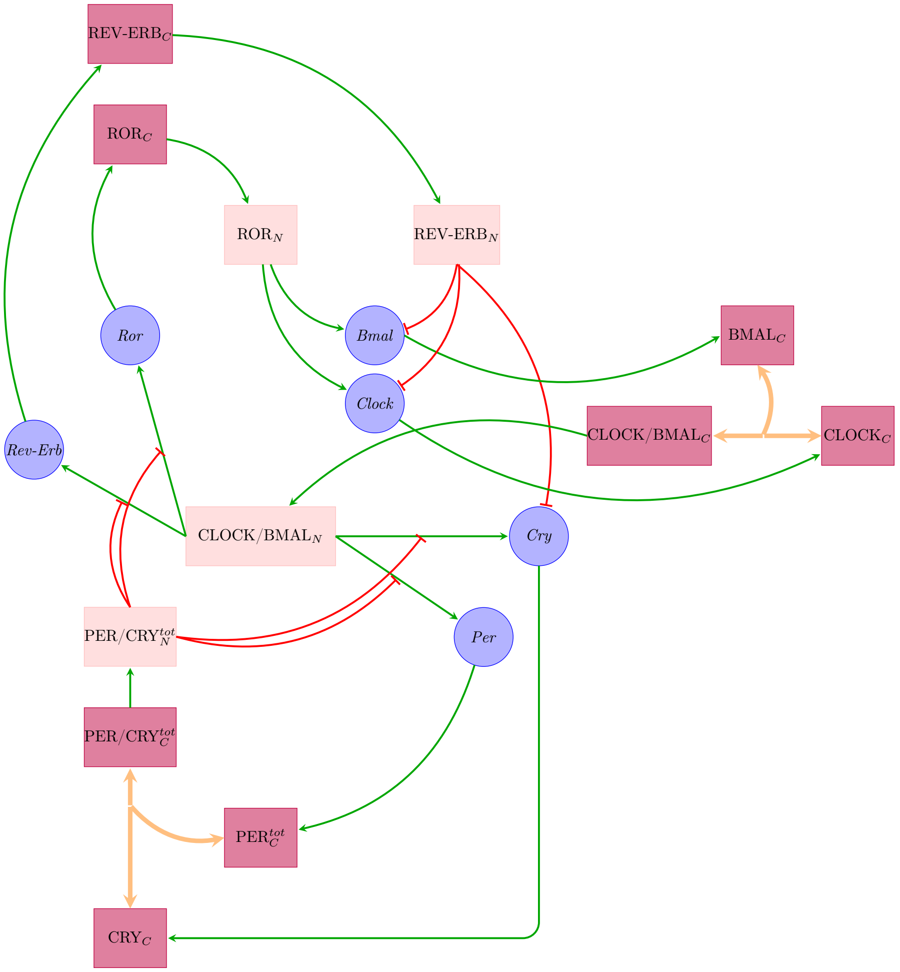

Figure S1-1: Mammalian cellular circadian clock model. Genes are represented by indigo colored circles, cytoplasmic proteins by pink rectangles and nuclear proteins by burgundy rectangles. Genes names are written in italic, Protein names in capital letters. Orange arrows denote complexation/decomplexation mechanisms.

1 Mathematical Description of the Quantitative Core-Clock Model

1.1 Variable Names

Variable name	Species Name
x_1	CLOCK/BMAL_N
x_2	PER/CRY_N^{tot}
x_5	REV-ERB_N
x_6	ROR_N
y_1	*Per*
y_2	*Cry*
y_3	*Rev-Erb*
y_4	*Ror*
y_5	*Bmal*
y_6	*Clock*
z_1	CRY_C
z_2	PER_C^{tot}
z_4	PER/CRY_C^{tot}
z_5	CLOCK_C
z_6	REV-ERB_C
z_7	ROR_C
z_8	BMAL_C
z_9	CLOCK/BMAL_C

Table 1: List of state variables of the model

1.2 Model equations

CLOCK/BMAL_C

$$\frac{dz_9}{dt} = k_{f_{z_9}} z_8 z_5 + \frac{v_c}{v_n} k_{e_{x_1}} x_1 - \frac{v_c}{v_n} k_{i_{z_9}} z_9 - k_{d_{z_9}} z_9 \tag{1.1}$$

CLOCK/BMAL_N

$$\frac{dx_1}{dt} = k_{i_{z_9}} z_9 - k_{e_{x_1}} x_1 - d_{x_1} x_1 \tag{1.2}$$

CLOCK_C

$$\frac{dz_5}{dt} = k_{p6} y_6 + k_{d_{z_9}} z_9 - k_{f_{z_9}} z_8 z_5 - d_{z_5} z_5 \tag{1.3}$$

Rev-Erb

$$\frac{dy_3}{dt} = V_{3_{\max}} \frac{1 + g \left(\frac{x_1}{k_{t_3}}\right)^b}{1 + \left(\frac{x_2}{k_{i_3}}\right)^c \left(\frac{x_1}{k_{t_3}}\right)^b + \left(\frac{x_1}{k_{t_3}}\right)^b} - d_{y_3} y_3 \tag{1.4}$$

Ror

$$\frac{dy_4}{dt} = V_{4_{\max}} \frac{1 + h\left(\frac{x_1}{k_{t_4}}\right)^b}{1 + \left(\frac{x_2}{k_{i_4}}\right)^c \left(\frac{x_1}{k_{t_4}}\right)^b + \left(\frac{x_1}{k_{t_4}}\right)^b} - d_{y_4} y_4 \tag{1.5}$$

REV-ERB$_C$

$$\frac{dz_6}{dt} = k_{p_3} y_3 - \frac{v_c}{v_n} k_{i_{z_6}} z_6 - d_{z_6} z_6 \tag{1.6}$$

ROR$_C$

$$\frac{dz_7}{dt} = k_{p_4} y_4 - \frac{v_c}{v_n} k_{i_{z_7}} z_7 - d_{z_7} z_7 \tag{1.7}$$

REV-ERB$_N$

$$\frac{dx_5}{dt} = k_{i_{z_6}} z_6 - d_{x_5} x_5 \tag{1.8}$$

ROR$_N$

$$\frac{dx_6}{dt} = k_{i_{z_7}} z_7 - d_{x_6} x_6 \tag{1.9}$$

Clock

$$\frac{dy_6}{dt} = V_{6_{\max}} \frac{1 + j\left(\frac{x_6}{k_{t_6}}\right)^b}{1 + \left(\frac{x_5}{k_{i_6}}\right)^c + \left(\frac{x_6}{k_{t_6}}\right)^b} - d_{y_6} y_6 \tag{1.10}$$

Bmal

$$\frac{dy_5}{dt} = V_{5_{\max}} \frac{1 + i\left(\frac{x_6}{k_{t_5}}\right)^b}{1 + \left(\frac{x_5}{k_{i_5}}\right)^c + \left(\frac{x_6}{k_{t_5}}\right)^b} - d_{y_5} y_5 \tag{1.11}$$

BMAL$_C$

$$\frac{dz_8}{dt} = k_{p_5} y_5 + k_{d_{z_9}} z_9 - k_{f_{z_9}} z_8 z_5 - d_{z_8} z_8 \tag{1.12}$$

Per

$$\frac{dy_1}{dt} = V_{1_{\max}} \frac{1 + a\left(\frac{x_1}{k_{t_1}}\right)^b}{1 + \left(\frac{x_2}{k_{i_1}}\right)^c \left(\frac{x_1}{k_{t_1}}\right)^b + \left(\frac{x_1}{k_{t_1}}\right)^b} - d_{y_1} y_1 \tag{1.13}$$

Cry

$$\frac{dy_2}{dt} = V_{2_{\max}} \frac{1 + d\left(\frac{x_1}{k_{t_2}}\right)^e}{1 + \left(\frac{x_2}{k_{i_2}}\right)^f \left(\frac{x_1}{k_{t_2}}\right)^e + \left(\frac{x_1}{k_{t_2}}\right)^e} \frac{1}{1 + \left(\frac{x_5}{k_{i_{21}}}\right)^{f_1}} - d_{y_2} y_2 \tag{1.14}$$

CRY_C

$$\frac{dz_1}{dt} = k_{p_2} y_2 + k_{d_{z_4}} z_4 - k_{f_{z_4}} z_1 z_2 - d_{z_1} z_1 \tag{1.15}$$

PER_C^{tot}

$$\frac{dz_2}{dt} = k_{p_1} y_1 + k_{d_{z_4}} z_4 - k_{f_{z_4}} z_1 z_2 - d_{z_2} z_2 \tag{1.16}$$

$\mathrm{PER/CRY}_C^{tot}$

$$\frac{dz_4}{dt} = k_{f_{z_4}} z_1 z_2 + \frac{v_c}{v_n} k_{e_{x_2}} x_2 - \frac{v_c}{v_n} k_{i_{z_4}} z_4 - k_{d_{z_4}} z_4 \tag{1.17}$$

$\mathrm{PER/CRY}_N^{tot}$

$$\frac{dx_2}{dt} = k_{i_{z_4}} z_4 - k_{e_{x_2}} x_2 - d_{x_2} x_2 \tag{1.18}$$

1.3 Model parameters

Parameter	Name	Value
**Degradation rates for nuclear proteins or nuclear protein complexes [hour^{-1}]**		
d_{x_1}	CLOCK/BMAL	0.1621
d_{x_2}	PER/CRY$_N^{tot}$	0.8199
d_{x_5}	REV-ERB$_N$	2.3792
d_{x_6}	ROR$_N$	2.492
**Degradation rates for mRNAs [hour^{-1}]**		
d_{y_1}	*Per*	2.0379
d_{y_2}	*Cry*	2.4048
d_{y_3}	*Rev-Erb*	2.9842
d_{y_4}	*Ror*	0.5863
d_{y_5}	*Bmal*	2.4876
d_{y_6}	*Clock*	0.0775
**Degradation rates for cytoplasmic proteins [hour^{-1}]**		
d_{z_1}	CRY$_C$	2.5
d_{z_2}	PER$_C$	0.0306
d_{z_5}	CLOCK$_C$	1.7965
d_{z_6}	REV-ERB$_C$	0.5052
d_{z_7}	ROR$_C$	0.2873
d_{z_8}	BMAL$_C$	1.0173
**Reaction rates for complex formation/dissociation**		
$k_{f_{z_9}}$	CLOCK/BMAL$_C$-complex formation **[nmol**$\times L^{-1}\times$**hours^{-1}]**	0.0032
$k_{d_{z_9}}$	CLOCK/BMAL$_C$-complex dissociation **[hours^{-1}]**	0.401
$k_{f_{z_4}}$	PER/CRY$_C^{tot}$-complex formation **[nmol**$\times L^{-1}\times$**hours^{-1}]**	0.0085
$k_{d_{z_4}}$	PER/CRY$_C^{tot}$-complex dissociation **[hours^{-1}]**	0.0001
**Transcription rates [nmol$\times L^{-1}\times$ hours^{-1}]**		
$V_{1_{\max}}$	*Per*	14.4768
$V_{2_{\max}}$	*Cry*	88.2565
$V_{3_{\max}}$	*Rev-Erb*	0.7015
$V_{4_{\max}}$	*Ror*	0.1342
$V_{5_{\max}}$	*Bmal*	3.0126
$V_{6_{\max}}$	*Clock*	0.0514
**Activation/inhibition rates [nmol$\times L^{-1}$]**		
k_{t_1}	*Per*-activation rate	1.5696
k_{i_1}	*Per*-inhibition rate	0.0357
k_{t_2}	*Cry*-activation rate	38.1838
k_{i_2}	*Cry*-inhibition rate	0.0073
$k_{i_{21}}$	*Cry*-inhibition rate	1.9219
k_{t_3}	*Rev-Erb*-activation rate	1.4549
k_{i_3}	*Rev-Erb*-inhibition rate	1.1548
k_{t_4}	*Ror*-activation rate	2.2668
k_{i_4}	*Ror*-inhibition rate	0.0051
k_{t_5}	*Bmal*-activation rate	45.3584
k_{i_5}	*Bmal*-inhibition rate	0.8345
k_{t_6}	*Clock*-activation rate	0.1244
k_{i_6}	*Clock*-inhibition rate	1.154
**Transcription fold activation (dimensionless)**		
a	*Per*	4.5093
d	*Cry*	33.1242
g	*Rev-Erb*	22.5318
h	*Ror*	8.6805
i	*Bmal*	12
j	*Clock*	4.2613
**Production rates [molecules $\times$ mRNA$^{-1}\times$ hour^{-1}]**		

k_{p_1}	PER_C^{tot}	309.2465
k_{p_2}	CRY_C	82.3894
k_{p_3}	REV-ERB_C	377.4519
k_{p_4}	ROR_C	783.2351
k_{p_5}	BMAL_C	2324.5261
k_{p_6}	CLOCK_C	9.8721
	**Import/Export rates [hour^{-1}]**	
$k_{i_{z_4}}$	PER/CRY_C^{tot}	0.0303
$k_{i_{z_6}}$	REV-ERB_C	0.0303
$k_{i_{z_7}}$	ROR_C	0.0538
$k_{i_{z_9}}$	CLOCK/BMAL_C	0.0024
	**Hill coefficients of transcription (dimensionless)**	
b	activation	1.2111
c	inhibition	7.8523
e	*Cry*-activation	4.1967
f	*Cry*-inhibition	7.6312
f_1	*Cry*-inhibition	7.9957
	**Volume proportion (dimensionless)**	
v_c	cytoplasm	0.93
v_n	nucleus	0.07

Table 2: List of parameters and estimated values.
