# Supplementary material for: Model learning to identify systemic regulators of the peripheral circadian clock
Source: Bioinformatics. 2021 Jul 12;37(Suppl 1):i401–9. doi: 10.1093/bioinformatics/btab297 (PMC8557835; doi:10.1093/bioinformatics/btab297)
Supplement: btab297_Supplementary_Data [file btab297_supplementary_data.zip › MARTINELLI.116.sup.2.pdf]

Supplementary File S2: parameter estimation and figures

Julien Martinelli, Sandrine Dulong, Xiao-Mei Li, Michèle Teboul,
Sylvain Soliman, Francis Lévi, François Fages, Annabelle Ballesta

1 Parameter estimation

Calibration of the cellular clock model against MMH-D3 hepatocytes mRNA expression was performed using the evolutionary algorithm CMA-ES (Hansen and Ostermeier, 2001), considered state of the art for parameter optimization in settings where gradient information cannot be easily accessed, typically in systems biology. The cost function was defined as the sum of squares between the time-resolved mRNA expression and the model simulated timecourses.

Exhaustive model search with linear regression was performed using the Scikit-learn package in Python (Pedregosa *et al.*, 2011). Computations were done on a laptop with a 2.9GHz Intel core I5 dual core.

References

Hansen, N. and Ostermeier, A. (2001). Completely derandomized self-adaptation in evolution strategies. *Evolutionary Computation*, **9**(2), 159–195.

Pedregosa, F., Varoquaux, G., Gramfort, A., Michel, V., Thirion, B., Grisel, O., Blondel, M., Prettenhofer, P., Weiss, R., Dubourg, V., Vanderplas, J., Passos, A., Cournapeau, D., Brucher, M., Perrot, M., and Duchesnay, E. (2011). Scikit-learn: Machine learning in Python. *Journal of Machine Learning Research*, **12**, 2825–2830.

2 Supplementary Pipeline

Pipeline 1: Shapley values computation.

Result: Shapley values summed over classes and trajectories

initialization $\boldsymbol{\phi} = 0_{\mathbb{R}^{10\times(N-1)}}$

for *all sets of regulator indices* $S \in I$ **do**

  for $c = 1, \ldots, 4$ **do**

    for $k = 1, \ldots, n$ **do**

      $\boldsymbol{\beta}_k^{(c)} = 0_{\mathbb{R}^{10}}$

      $\boldsymbol{\beta}_{k,S}^{(c)} \leftarrow \underset{\boldsymbol{\beta}_{k,S}^{(c)}}{\operatorname{argmin}}\ \ell(y_k^{(c)}, \bar{\boldsymbol{z}}_S^{(c)}, \boldsymbol{\beta}_{k,S}^{(c)})$

      for $i = 1, \ldots, N-1$ **do**

        $\phi_S(t_i) \leftarrow \phi_S(t_i) + |\boldsymbol{\beta}_{k,S}^{(c)}\ \bar{z}_S^{(c)}(t_i)|$

$\boldsymbol{\phi} \leftarrow \dfrac{\boldsymbol{\phi}}{4n\#I}$

3 Supplementary figures

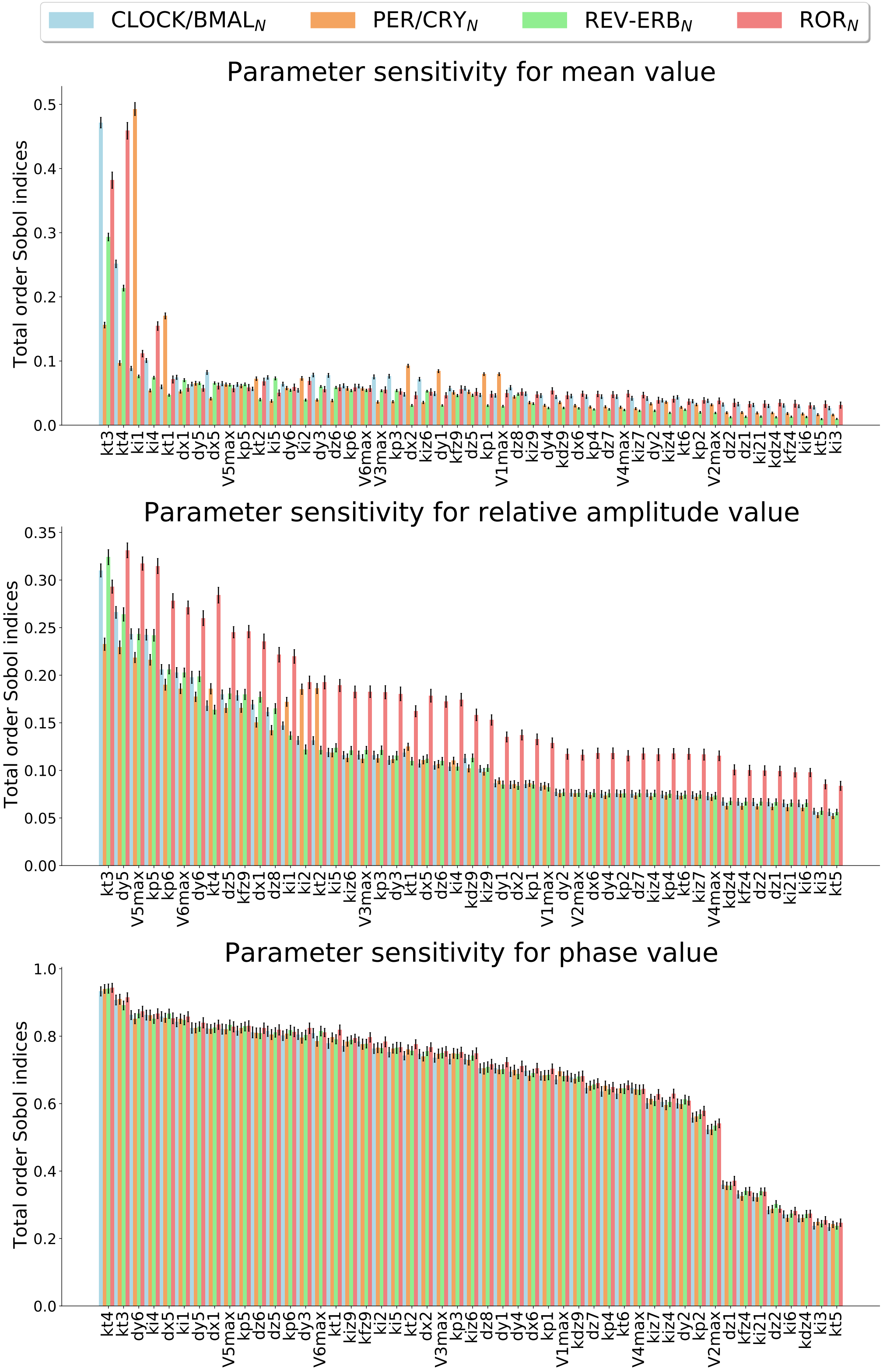

Figure S2-1: Sensibility analysis performed on the mean, relative amplitude and phase of the modulatory variables of the *in vitro* circadian clock model. Total order sobol indices plotted, with estimated standard deviations. Parameter definitions provided in supplementary file S1.

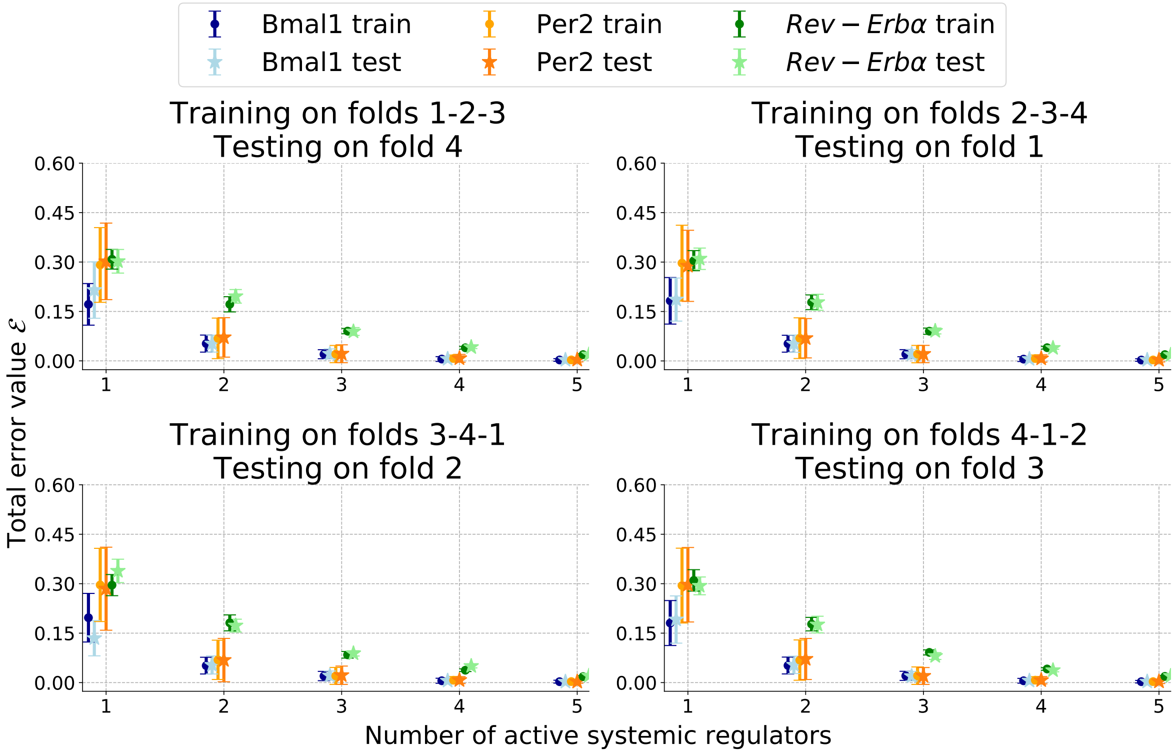

Figure S2-2: For each gene, the total error of the best-fitting model depending on its number of nonzero terms is reported under **(H1-H3)**. Timepoints were shuffled and divided in 4 folds on which 4-fold cross validation was performed.

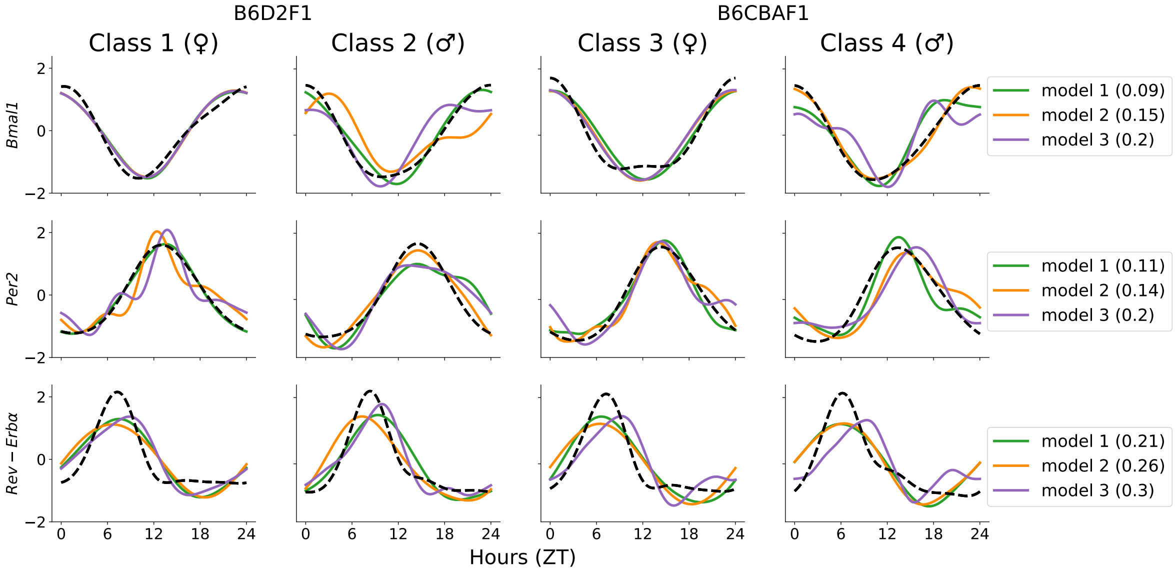

Figure S2-3: Fit of one residual trajectory (dashed black curves) for each gene and each class. 3 different models (solid colored curves) are used for the sake of error visualization.

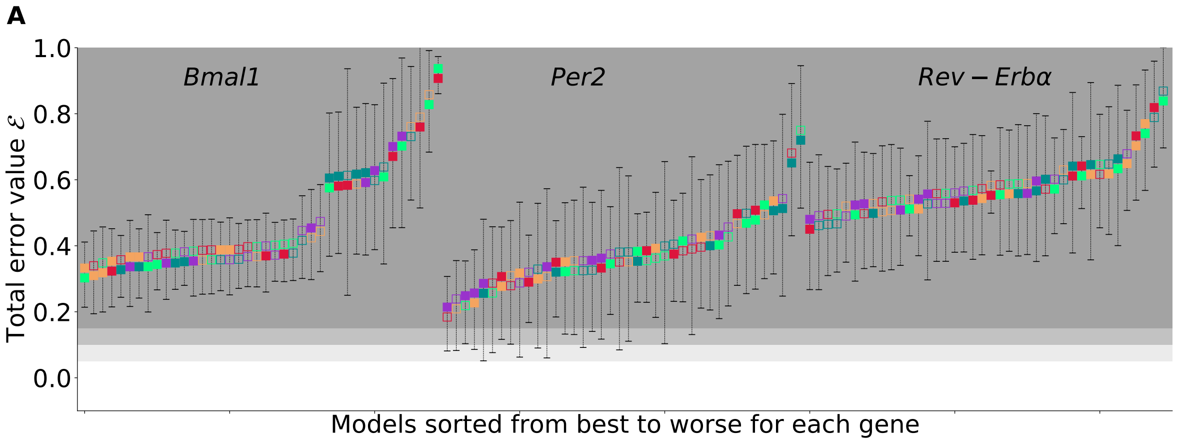

Figure S2-4: Total error for each of the 40 2-terms models, for all genes. Mean and standard deviation were computed across residual trajectories under **(H2-H3)**. Colored squares at the mean describe the regulators involved for each model, with the top square referring to the dominant regulator. Areas defined by different shades of gray refer to thresholds of total error equal to 0.05, 0.1 and 0.15.

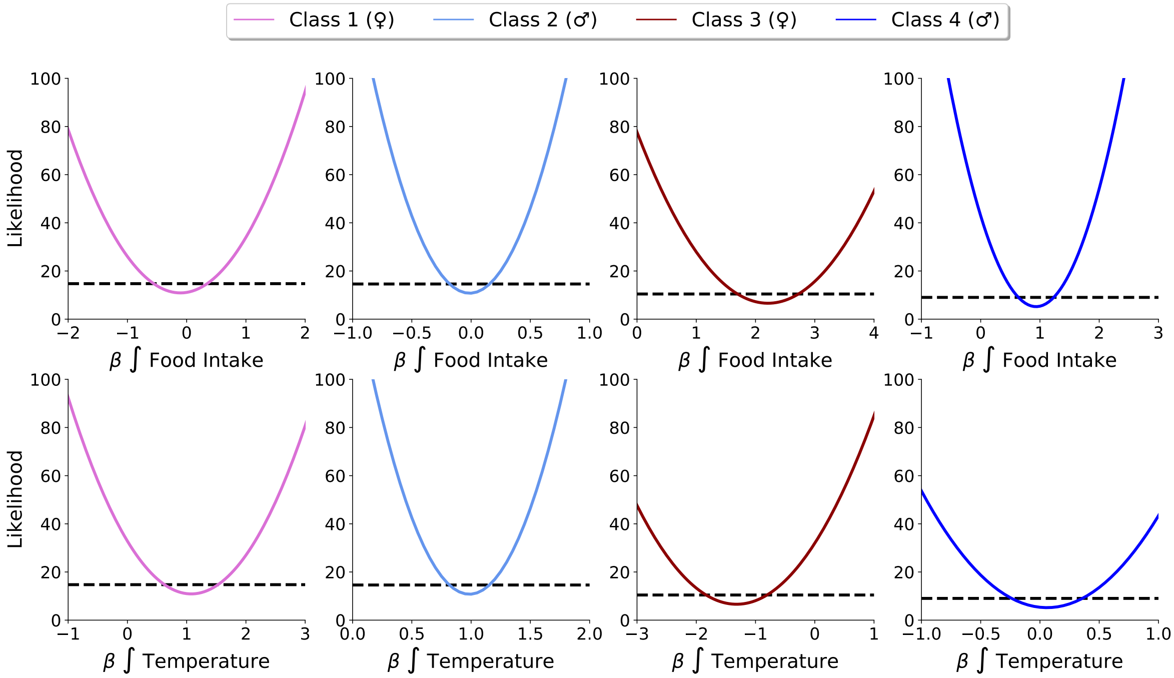

Figure S2-5: Profile likelihood obtained for *Bmal1* best 2-term model under **(H1-H3)**. Black dashed lines delimit the 95% confidence interval in which the parameters are identifiable.

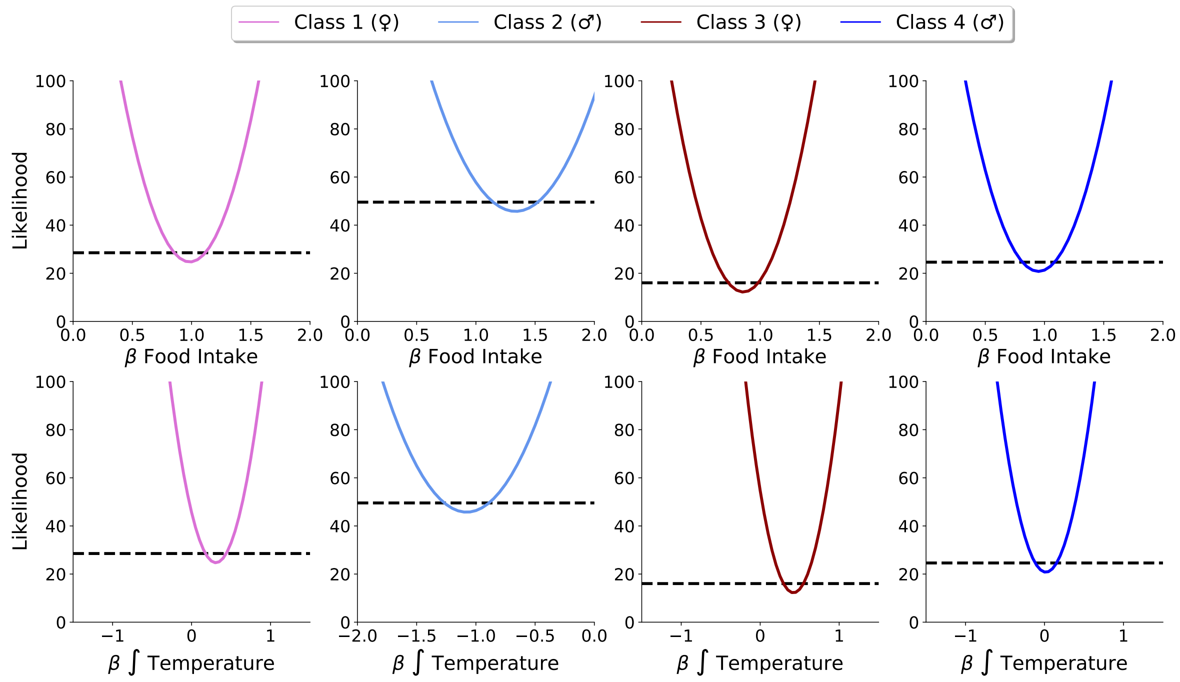

Figure S2-6: Profile likelihood obtained for *Per2* best 2-term model under **(H1-H3)**. Black dashed lines delimit the 95% confidence interval in which the parameters are identifiable.

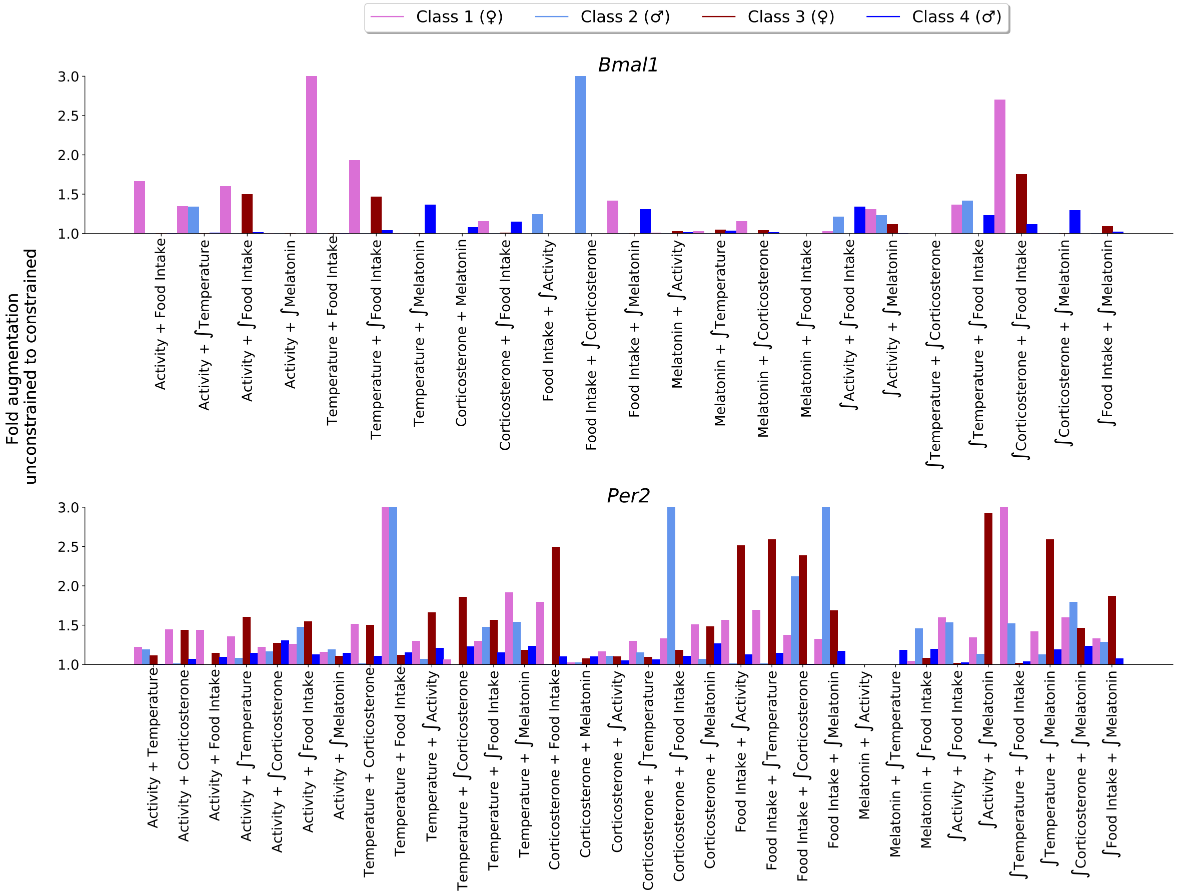

Figure S2-7: Under **(H1-H3)**, for each 2-term model whose unconstrained optimal parameters did not meet the classwise sign equality constrain, the fold augmentation between the total error in the unconstrained case versus the constrained case is plotted. Only classes where the sign of at least one regulator was modified from unconstrained to constrained optimization are plotted.
